# Cellulose Nanofiber/Carbon Nanotube Conductive Nano-Network as a Reinforcement Template for Polydimethylsiloxane Nanocomposite

**DOI:** 10.3390/polym10091000

**Published:** 2018-09-07

**Authors:** Chuchu Chen, Xiangting Bu, Qian Feng, Dagang Li

**Affiliations:** College of Materials Science and Engineering, Nanjing Forestry University, Nanjing 210037, China; n150407127@njfu.edu.cn (X.B.); njfu_xx@njfu.edu.cn (Q.F.)

**Keywords:** cellulose nanofiber, polydimethylsiloxane, nanocomposite, mechanical properties, electrical conductivity

## Abstract

Both cellulose nanofiber (CNF) and carbon nanotube (CNT) are nanoscale fibers that have shown reinforcing effects in polymer composites. It’s worth noting that CNF and CNT could form a three-dimensional nano-network via mixing and vacuum filtration, which exhibit excellent mechanical strength and electrical conductivity. In this study, the developed CNF/CNT film was applied as a nano-network template and immersed into polydimethylsiloxane (PDMS) solutions. By controlling the immersed polydimethylsiloxane pre-polymer concentration, the PDMS/CNF/CNT nanocomposite with various PDMS contents were fabricated after a curing process. Morphological images showed that the CNF/CNT nano-network was well-preserved inside the PDMS, which resulted in significantly improved mechanical strength. While increasing the PDMS content (~71.3 wt %) gave rise to decreased tensile strength, the PDMS-30/CNF/CNT showed a fracture strain of 7.5%, which was around seven fold higher than the rigid CNF/CNT and still kept a desirable strength—Young’s modulus and conductivity of 18.3 MPa, 805 MPa, and 0.8 S/cm, respectively. Therefore, with the enhanced mechanical properties and the electrical conductivity, the prepared PDMS/CNF/CNT composite films may offer promising and broad prospects in the field of flexible devices.

## 1. Introduction

The rapid development of flexible electronic devices places a high demand for high-performance conductive base-materials. The challenges currently remaining are to develop such materials providing both high mechanical and electrical properties. Polydimethylsiloxane (PDMS) is a widely used polymer in various applications, including wearable/implantable devices and microfluidics, owing to its biocompatibility, optical transparency, flexibility, and elasticity [1,2,3]. Recently, PDMS has attracted considerable attention as a soft component polymer to be utilized as base-materials for flexible devices [4,5,6,7,8]. In such flexible base-materials, micro or nano-sized structures, composed of multi-reinforcing functions, e.g., high electrically conductive, or mechanical strength, are essential to further expand its applications [9,10,11,12]. Cellulose, a linear chain polysaccharide consisting of repeated β-(1→4)-d-glucopyranose units, makes its nano-fibrillated form (cellulose nanofiber) a desirable reinforcing material for polymer nanocomposites [13,14,15,16,17,18]. One of the typical methods to combine polymer with reinforcing nanoparticles is a solution-mixing and casting process. For example, by mixing CNF with a polymer solution, followed with polymerization, a CNF-based nanocomposite with improved mechanical properties, could be fabricated, but with a limited CNF content [19,20,21]. Carbon nanotubes (CNTs, which was highly conductive) with loading percentages around 2–8 wt % is incorporated into a PDMS polymer to prepare a conductive PDMS/CNT nanocomposite via mechanical stirring under chloroform solutions [22]. The main challenge in fabricating nanofiber-based composites is their well-dispersion in the polymer matrix and the broad range of composition controls [23,24], several attempted methods have been reported. By layer-by-layer curing, PDMS elastomer and rigid cellulose nanocrystals are covalently bonded, possessing a sandwiched structure [25]. To increase the CNT content in the PDMS, CNTs are produced into a film via vacuum filtration and then immersed into PDMS solutions to prepare flexible PDMS/CNT composite films. Such immersion processes provide the composite higher CNT loading fillers and show excellent electrical conductivity, but with undesirable mechanical strength due to the nanofiber aggregation [26].

In this study, CNF/CNT film was prepared via mixing and vacuum filtration. Since the well-dispersed CNF in water could be used as a dispersing agent for CNT and afforded to form a rigid nano-network structure [27], the CNF/CNT film was served as a template to develop nanocomposite via the immersion process. In this process, the CNF/CNT nano-networks provided the composite with both improved mechanical strength and electrical conductivity due to the nano-network structures and the continuous electronic transmittance pathways in the nanocomposite. Specifically, by controlling the PDMS pre-polymer concentration during the immersion process, the PDMS/CNF/CNT composite films, with various PDMS contents (in a broad range from 23.7 to 71.3 wt %), were fabricated after curing. Afterwards, morphological features, chemical bonding interactions, tensile properties, and electrical conductivity were further discussed.

## 2. Materials and Methods

### 2.1. Materials

The silicone elastomer (Sylgard 184) of polydimethylsiloxane (PDMS) and curing agents were purchased from the company of Dow Corning (Midland, MI, USA). The PDMS was formed by mixing the liquid pre-polymer (Sylgard 184 A) and the curing agent (Sylgard 184 B) at a ratio of 10:1 and heated at 60 °C. The Multi-wall carbon nanotube (CNT) paste (15–20 nm diameter, 5–15 μm length, 97.5 wt % purity) were purchased from Shenzhen Nanotech-Port Co., Ltd., Shenzhen, China. The CNT content was 5 wt % (in water). Wood powder from the softwood Hinoki cypress (Chamaecyparis obtusa) was used as the raw material and was purified as follows [28]. The lignin was removed using an acidified sodiumchlorite (NaClO_2_) solution at 90 °C for 1 h and this treatment was repeated four times. Then, an alkaline treatment with 6 wt % potassium hydroxide (KOH) at 80 °C for 2 h was performed to remove the hemicellulose. After each chemical treatment, the samples were filtered and rinsed with distilled water until the residues were neutralized. Finally, the purified sample was kept in a water-swollen state without drying, and had a concentration around 1 wt %. Sodium hydroxide, sodiumchlorite, potassium hydroxide, and *n*-hexane solutions were all of laboratory grade and were used as received.

### 2.2. Preparation of CNF/MWCNT Film

1 wt % purified cellulose slurry was passed through a grinder (MKCA6-2; Masuko Corp., Tokyo, Japan) twice at 1500 rpm. The grinding treatment was performed with a clearance gauge of −2.5 (corresponding to a 0.25 mm shift) from the zero position, which was determined as the point of slight contact between the two grinding stones [28]. The obtained cellulose nanofiber (CNF) suspension was mixed with CNT at a solid weight ratio of 1:1 and diluted using distilled water into the concentration of 0.1 wt %. The mixture (CNF/CNT) was then ultra-sonicated in an ultrasonic wave cell crusher (XO-1200, Xianou Biological Technology Co. Ltd., Nanjing, China) for 20 min (960 W, 50 Hz) to give a black homogeneous dispersion and vacuum-filtered into a wet CNF/CNT film using a polytetrafluoroethylene membrane (PTFE, 0.2 μm) filter. The resulting wet CNF/CNT film was immersed into *n*-hexane (6 h each time, 4 times) by solvent exchange to remove the water inside the film.

### 2.3. Preparation of PDMS/CNF/MWCNT Film

Liquid PDMS pre-polymer was diluted using *n*-hexane under different weight concentration of 1, 3, 5, 10, 20, and 30 wt % (20 g in total). The *n*-hexane solvent-exchanged CNF/CNT film was then immersed into the above liquid PDMS pre-polymer (base), respectively, for 12 h at room temperature. Afterward, the curing agent (base: curing agent = 10: 1 *w/w*) was slowly added into the PDMS pre-polymer solutions. After 0.5 h, the CNF/CNT film was taken out and slightly removed the redundant PDMS solutions on the surface using filter paper. The as-prepared film sample was orderly covered by a PTFE (1 mm) and a glass plate on each side and was cured at 60 °C for 2 h to give a solid PDMS/CNF/CNT composite film with different PDMS contents. The synthesized PDMS/CNF/CNT composite samples were denoted as PDMS-*n*/CNF/CNT, where *n* represents the PDMS pre-polymer concentration (*n* = 1, 3, 5, 10, 20, and 30). The neat PDMS film was fabricated by pouring the PDMS solutions (base: curing agent = 10: 1 *w/w*) into Teflon Petri dishes and cured at the 60 °C for 2 h.

### 2.4. Characterization

The surface morphology of the samples were characterized using a field emission scanning electron microscope (FE-SEM, HITACHI SU-8010, Hitachi, Tokyo, Japan), operating at 1.5 kV. Transmission electron microscope (TEM) (JEM-2100, JEOL Inc, Tokyo, Japan) was used to investigate the morphology of CNF/CNT mixture suspension. One drop of diluted suspension was put on carbon-coated copper grid and then dried for TEM analysis. The X-ray diffraction (XRD) patterns were recorded on a Rigaku X-ray diffractometer (Smartlab-3kw; Rigaku Corp., Tokyo, Japan) using Cu-Kα radiation (40 kV and 40 mA), with a scanning speed of 5 deg/min. Fourier-transform infrared spectroscopy was performed using FTIR spectrometer (PerkinElmer, Inc., Waltham, MA, USA) equipped with a universal attenuated total reflectance accessory. The sample was scanned 16 times from 500 to 4000 cm^−1^ with a resolution of 4 cm^−1^. The tensile properties of the pure PDMS and nanocomposite samples were investigated by using a universal material-testing machine (SANS, Shenzhen Co. Ltd., Shenzhen, China) at room temperature. The sample was cut (in 35 mm length, 5 mm width) and test with a cross-head speed of 10 mm/min. The average value of the tensile stress, fracture strain, and Young’s modulus was calculated for at least five specimens [4,26]. Electrical conductivity of the PDMS/CNF/CNT composite films were measured at room temperature using a four-point probe working station (RTS-8, Probes Tech, Guangzhou, China) after calibration. The thickness and diameter of the samples were measured before testing and the average value of the conductivity was calculated for at least three specimens.

## 3. Results and Discussion

**Figure 1:**
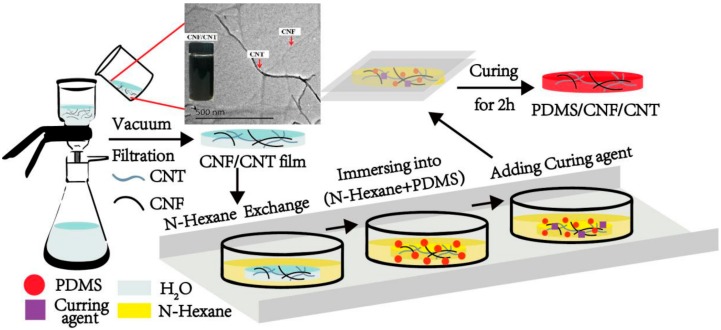
Preparation route of Polydimethylsiloxane/cellulose nanofiber/carbon nanotube (PDMS/CNF/CNT) nanocomposite and the TEM image of CNF/CNT mixture suspension (the inserted digital image of CNF/CNT dispersion exhibited uniform appearance even after two months).

Previously, we reported that the well-dispersed CNF in water could act as a dispersing agent for CNT and form a three-dimensional conductive network after mixing and vacuum-filtration. In particular, when CNF and CNT performed a ratio at 1:1 (*w*/*w*), the obtained CNF/CNT film showed desirable electrical conductivity due to the well-dispersion of CNTs [27]. As shown in Figure 1, the resulting CNF/CNT black dispersion displays an uniform appearance even after 2 months. The inserted TEM image exhibits the well-dispersed nanofibers without any significant aggregation (with the CNF diameter around 20–30 nm). This phenomenon was due to both the short-range hydrophobic interactions between the CNTs and the specific crystalline faces (hydrophobic (200) planes) of the cellulose, and the long-range electrostatic repulsion between the sulfated cellulose provided the stabilization of the CNF/CNT dispersion [29]. To improve the flexibility of the conductive film, the PDMS elastomer was introduced into the film, where the CNF/CNT served as a conductive network template. The fabrication route of the PDMS/CNF/CNT composite film via the immersion approach is shown in Figure 1. As described, CNF was mixed with CNT and formed into a wet film (containing three-dimensional nano-networks) via vacuum-filtration. Since PDMS is hydrophobic elastomer (soluble in *n*-hexane), it (PDMS pre-polymer) can not penetrate into the wet CNF film in the water system. Therefore, the resulting wet CNF was dehydrated by solvent exchange (using *n*-hexane), where the *n*-hexane replaced the inside water to facilitate the following immersion process. Afterwards, the CNF/CNT film was immersed into PDMS pre-polymer solution to allow the PDMS to penetrate or fill inside the CNF/CNT porous nano-networks. To prevent the PDMS from self-polymerization at room temperature, a curing agent was sequentially added into the mixture (maintained for 30 min) and then heated at 60 °C for 2 h to quickly complete the curing process of the PDMS. Finally, the PDMS/CNF/CNT (with various PDMS content) was fabricated. The PDMS content of the composite was calculated based on the original weight of the CNF/CNT and the weight of the prepared PDMS/CNF/CNT nanocomposite. As calculated, PDMS content in each PDMS-*n*/CNF/CNT composite sample (*n* = 1, 3, 5, 10, 20, and 30) was 23.7 ± 2.1%, 31.3 ± 1.6%, 42.5 ± 2.7%, 50.5 ± 1.2%, 59.0 ± 2.7%, and 71.3 ± 3.1%, respectively, where *n* represents the PDMS pre-polymer concentration. It seems that increasing the immersed concentration of PDMS pre-polymer resulted in the increased PDMS content in the final PDMS-*n*/CNF/CNT nanocomposite. In fact, we also controlled the immersed PDMS pre-polymer concentration of 40 and 50 wt %, whereas the final PDMS content in the nanocomposite cannot be further increased.

**Figure 2:**
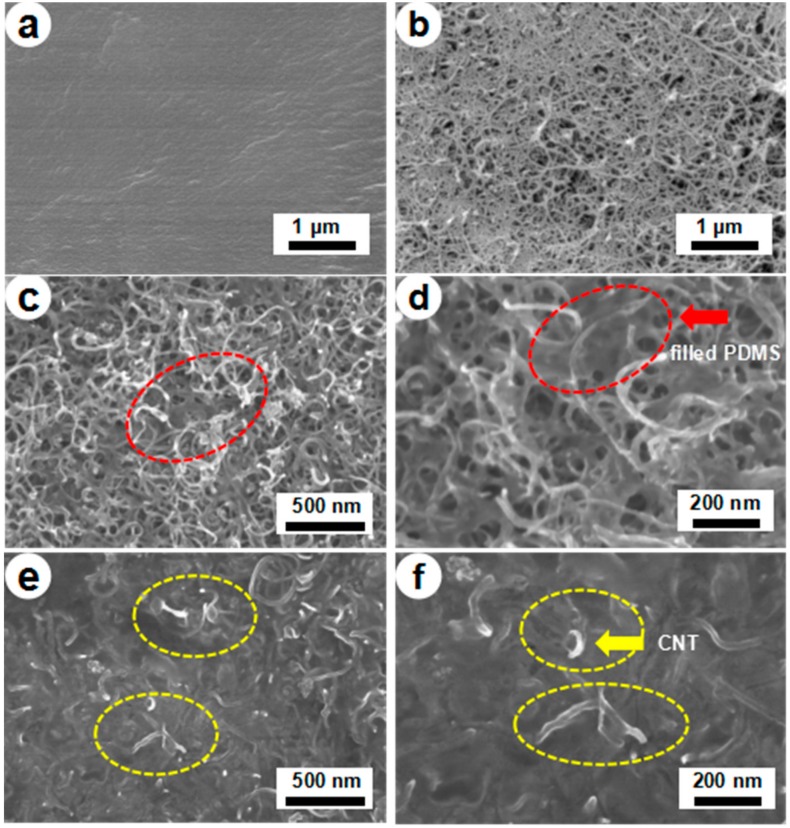
Field emission scanning electron microscope (FE-SEM) images of Pure PDMS (**a**) CNF/CNT (**b**) PDMS-1/CNF/CNT composite film (**c**) ×50,000 (**d**) ×100,000 and PDMS-30/CNF/CNT composite film (**e**) ×50,000 and (**f**) ×100,000.

Morphological features of the PDMS, CNF/CNT, and PDMS/CNF/CNT were characterized by Field emission scanning electron microscope (FE-SEM). It is clearly seen that PDMS (Figure 2a) shows a flat and smooth surface, whereas CNF/CNT (Figure 2b) exhibits a nano-network structure. Figure 2c–f shows the PDMS-1/CNF/CNT (PDMS content: 23.7 ± 2.1%) and the PDMS-30/CNF/CNT (PDMS content: 71.3 ± 3.1%) nanocomposite with a different PDMS content and magnified by 50,000 and 100,000 times, respectively. When PDMS was in low content, a nanofiber network structure was clearly observed in the nanocomposite (Figure 2c) without significant fiber aggregations. In the magnified image (Figure 2d), a glue-like substance was filled inside the pores of nano-networks (marked by red circles), which was considered as the elastomer PDMS [7,26]. While it is difficult to distinguish the CNF and CNT due to their similar fibrous shape, CNT is highly conductive and presents a highly bright color in the FE-SEM images (for example, marked by yellow circles in Figure 2. when the PDMS content was increased to 71.3% (PDMS-30/CNF/CNT), surface of the nanocomposite became smooth (without visible pores) and nano-network structures were indistinct inside the bulky polymers (PDMS). In the magnified picture (Figure 2e), compared to the PDMS-1/CNF/CNT sample, it seems that the bulky PDMS polymer fully filled inside the porous CNF/CNT nano-networks and exhibited a flat surface morphology whereas bright CNT endings are still seen inside the matrix. Thus, the results from FE-SEM characterization indicated that PDMS polymer filled inside the CNF/CNT nano-pores after polymerization, where the CNF/CNT template remained the nano-network structures providing continuous electronic transmission pathways in the composite films.

**Figure 3:**
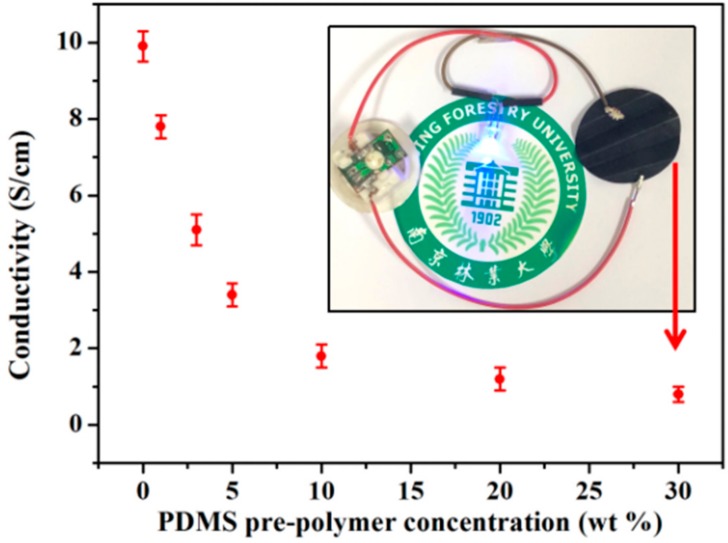
Conductivity of PDMS/CNF/CNT depending on the PDMS pre-polymer concentration during the immersion process, and a digital image which shows that the folded PDMS-30/CNF/CNT was used as a wire to let a blue LED glow well (inserted picture).

Considering that the CNF/CNT was highly conductive (conductivity was around 9.9 S/cm at the ratio 1:1 *w*/*w*), the electrical performance of the composite film was investigated after combining with PDMS. As Figure 3 shows, when the immersed PDMS pre-polymer concentration increased from 1 to 10 wt %, conductivity of the resultant PDMA/CNF/CNT nanocomposite was dramatically decreased from 7.8 S/cm (PDMA-1/CNF/CNT) to 1.8 S/cm (PDMA-10/CNF/CNT). As reported, CNF and CNT could form three-dimensional conductive networks via mixing and vacuum-filtration, which provided continuous electronic pathways in the hybrid composite [27]. This decreased electrical conductivity was probably produced by the PDMS polymer which filled inside the nano-pores, avoiding a good electrical contact with the electrodes. Afterwards, conductivity of the PDMA/CNF/CNT was slightly reduced to 0.8 S/m (PDMA-30/CNF/CNT) when the PDMS content was further increased to 71.3 wt %. This PDMA-30/CNF/CNT nanocomposite was highly flexible (allowed rolled and folded) and can be used as a wire to let a blue LED glow well even with a high PDMS content, as shown in Figure 3.

**Figure 4:**
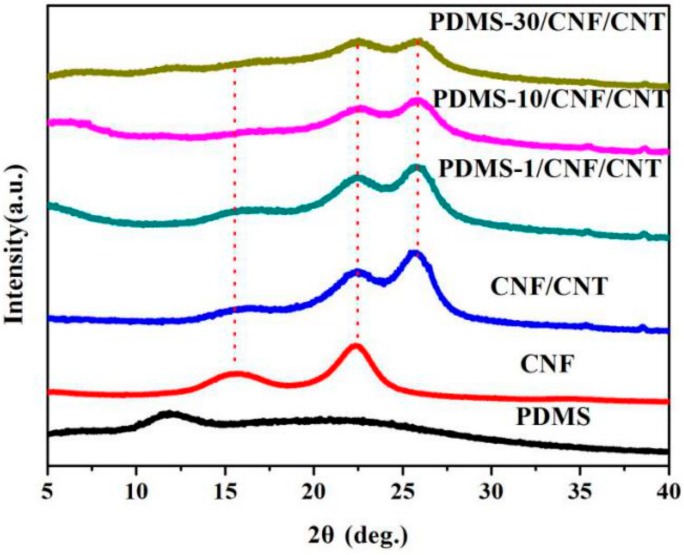
X-ray diffraction profiles of PDMS, CNF, CNF/CNT, and PDMS/CNF/CNT nanocomposite film samples.

**Figure 5:**
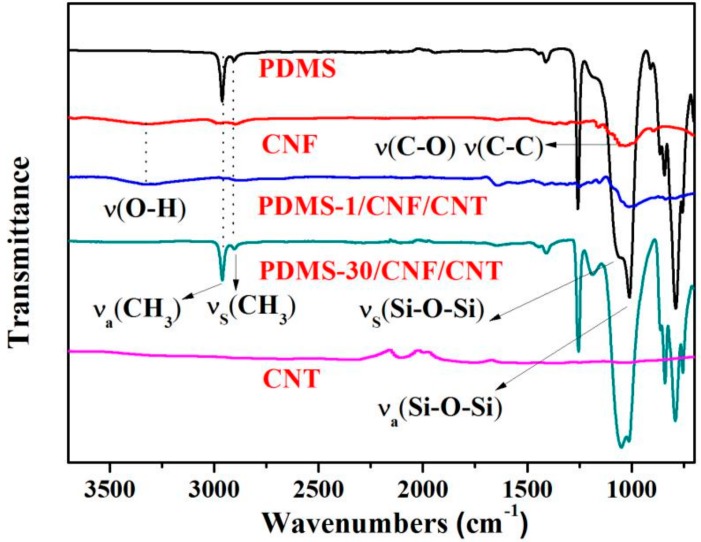
FTIR spectra of PDMS, CNF, CNT, and PDMS/CNF/CNT nanocomposite samples.

XRD measurements were performed to investigate the crystalline structure of the nanocomposite after combining CNF/CNT and PDMS. In Figure 4, CNF showed a typical cellulose I crystal form with characteristic peaks around 2θ = 16.5° and 22.5° which corresponds to the (010) and (110), respectively. Pure PDMS exhibited a wide diffraction peak between 2θ = 5°–40° (with a characteristic amorphous halo at 12°) [30], indicating that the PDMS was in an amorphous state [1]. Due to the symmetrical structure, it can rarely see any peaks in the FTIR spectrum of dried CNTs powders in Figure 5. The very weak peaks may be the absorption peaks of the instrument itself. After introducing PDMS into the CNF/CNT, all resulting PDMS/CNF/CNT samples kept the respective CNF characteristic peaks and the peaks specific to CNT appeared at 2θ of approximately 26° (as marked by red lines in Figure 5). With the increasing PDMS content, intensity of the characteristic peaks (CNF and CNT) was decreased, which indicated that some amorphous PDMS wrapped around the CNF and CNT nanofibers. While all the diffraction peak intensities decreased in the nanocomposite, both the original characteristic structure of cellulose and carbon nanotube were still observed in the composite, suggesting a well-incorporation between the hybrid components [31,32].

To investigate the interactions between the different components, PDMS, CNF, CNT, and PDMS/CNF/CNT nanocomposites samples were characterized by FTIR measurements, as shown in Figure 5. Pure PDMS displayed characteristic peaks at 2962 and 2904 cm^−1^, which can be assigned to CH_3_ asymmetric and symmetric stretching, respectively. The peak appearing at 1257 cm^−1^ is assigned to CH_3_ symmetric bond bending, and the peaks at 1064 and 1010 cm^−1^ are assigned to Si−O−Si symmetric and asymmetric band stretching, respectively. For the CNF, the main bands due to the cellulose spectrum are O−H stretching at around 3300 cm^−1^ and C−O and C−C stretching modes at 1018 cm^−1^ [33]. After combining PDMS with CNF/CNT, the obtained composite did not exhibit the formation of new or existing chemical bands relative to those of the original component. However, when the PDMS content in the composite film increased from 23.7 (PDMS-1/CNF/CNT) to 71.3 wt % (PDMS-30/CNF/CNT), the O−H stretching peak was weakened due to the formation of the hydrophobic PDMS layer over the nanofiber network. Furthermore, the Si−O−Si symmetric band shift from 1064 to 1049 cm^−1^ was probably modified via hydrogen bonding between the hydroxyl groups and the oxygen of the siloxane groups of the PDMS [34].

**Figure 6:**
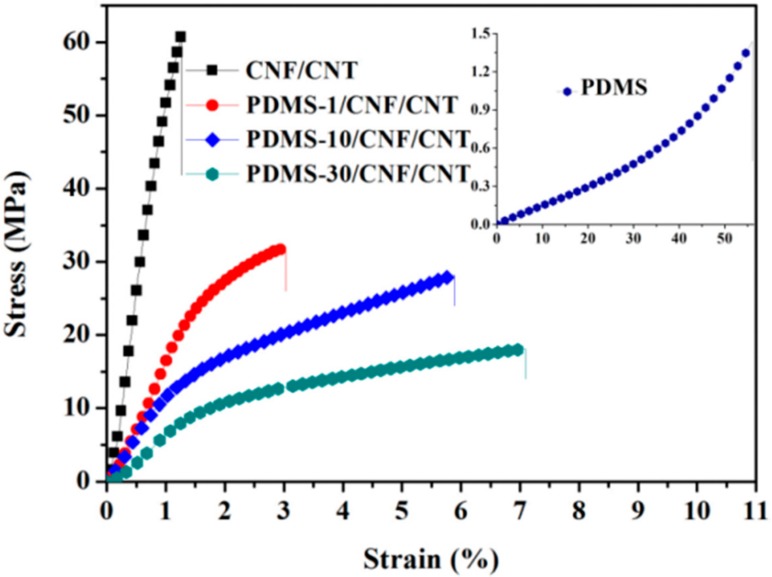
Tensile stress-strain curves of the CNF/CNT and PDMS/CNF/CNT composite film in different PDMS content.

As seen from the FE-SEM images above (Figure 2), CNF/CNT formed a homogeneous nano-network (acted as a template) where PDMS was filled inside the nano-pores or produced a thin layer covered on the surface (when increasing the PDMS content). Owing to the high-strength nano-network, tensile properties of the PDMS/CNF/CNT nanocomposite were investigated. In Figure 6, CNF/CNT film showed a rigid structure with a tensile strength, Young’s modulus, and a fracture strain of 61.7 MPa, 5132 MPa, and 1.1%, respectively. After incorporation with PDMS, the fracture strain of the composite films was increased due to the elastic PDMS. Notably, all PDMS/CNF/CNT composite samples exhibited significantly improved tensile strength and Young’s modulus, as compared to the neat PDMS. As shown in Figure 5 and Table 1, for the PDMS-1/CNF/CNT film sample, when the incorporated PDMS was around 23.7 wt %, tensile strength increased from 1.5 MPa (neat PDMS) to 32.6 MPa, which was almost 20 fold higher than the pure PDMS. Notably, Young’s modulus of the PDMS was significantly enhanced to 1616 from 1.4 MPa. Further increasing the PDMS content to 71.3 wt %, the resultant PDMS-30/CNF/CNT showed elastic performance with a fracture elongation and Young’s modulus of 7.3% and 805 MPa, respectively. These results indicated that by combining the rigid CNF/CNT nano-network template with the elastic PDMS, the formed PDMS/CNF/CNT composite film provided both improved elastic behavior and considerable tensile strength. While the increased PDMS caused the decreased conductivity, the developed PDMS-30/CNF/CNT (with the PDMS content of 71.3 wt %) maintained the conductivity of 0.8 S/cm. Therefore, by changing the immersed concentration of PDMS pre-polymer, the reinforcing nanofiber composition content could be controlled in a broad rang via the immersion method, leading to controllable mechanical properties for the resulting nanocomposites.

## 4. Conclusions

In this study, the PDMS/CNF/CNT nanocomposites were fabricated via the template-immersion method. With such a method, PDMS pre-polymer was controllably penetrated inside the porous CNF/CNT nano-networks (by adjusting the pre-polymer concentration) with the assistance of *n*-hexane solvent-exchange. Different from the traditional mixing and casting method, the immersion method gave the nanocomposite a broad composition range (PDMS content from 23.7 to 71.3 wt %). FE-SEM characterization indicated that the PDMS polymer filled inside the porous CNF/CNT nano-network after polymerization, resulting in significantly improved tensile properties (20 fold higher strength), compared to the neat PDMS (when the PDMS content was around 23.7 wt % in the PDMS-1/CNF/CNT composite film). Specifically, the developed nanocomposite exhibited considerable electrical conductivity ranging from 7.8 to 0.8 S/cm, even with the PDMS content increased from 23.7 to 71.3 wt %. Considering the desirable tensile performance and conductivity, this PDMS/CNF/CNT nanocomposite will have potential applications in flexible and electrical device fields.

## Figures and Tables

**Table 1:** Tensile properties of the PDMS, CNF/CNT, and PDMS/CNF/CNT nanocomposite.

Sample Data	PDMS-1/CNF/CNT	PDMS-10/CNF/CNT	PDMS-30/CNF/CNT	CNF/CNT	PDMS
Thickness (μm)	60 ± 3	74 ± 4	96 ± 4	41 ± 3	61 ± 2
PDMS (%)	23.7 ± 2.1	50.5 ± 2.2	71.3 ± 3.1	0.0	100.0
Stress (MPa)	32.6 ± 3.7	28.1 ± 1.6	18.3 ± 2.5	61.7 ± 3.1	1.5 ± 0.2
Strain (%)	3.1 ± 0.2	5.8 ± 0.5	7.3 ± 0.4	1.1 ± 0.2	51 ± 3
Young’s modulus (MPa)	1616 ± 114	1322 ± 107	805 ± 53	5132 ± 308	1.4 ± 0.2
